# Tocilizumab for juvenile idiopathic arthritis: a single-center case series

**DOI:** 10.1590/1516-3180.2018.0489220719

**Published:** 2020-03-06

**Authors:** Fatma Yazılıtaş, Semanur Özdel, Doğan Şimşek, Özlem Aydoğ, Evrim Kargın Çakıcı, Gökçe Gür Can, Tülin Güngör, Mehmet Bülbül

**Affiliations:** I MD. Physician and Pediatric Nephrologist, Department of Pediatric Nephrology, Dr. Sami Ulus Kadin Doğum Çocuk Sağliği ve Hastaliklari Eğitim ve Araştirma Hastanesi, Sağlik Bilimleri Üniversitesi, Ankara, Turkey.; II MD. Physician and Pediatric Rheumatologist, Department of Pediatric Rheumatology, Dr. Sami Ulus Kadin Doğum Çocuk Sağliği ve Hastaliklari Eğitim ve Araştirma Hastanesi, Sağlik Bilimleri Üniversitesi, Ankara, Turkey.; III MD. Physician and Pediatric Rheumatologist, Department of Pediatric Rheumatology, Dr. Sami Ulus Kadin Doğum Çocuk Sağliği ve Hastaliklari Eğitim ve Araştirma Hastanesi, Sağlik Bilimleri Üniversitesi, Ankara, Turkey.; IV MD. Physician, Professor, Pediatric Nephrologist and Rheumatologist, Department of Pediatric Nephrology and Rheumatology, Ondokuz Mayis Üniversitesi Tip Fakültesi, Samsun, Turkey.; V MD. Physician and Pediatric Nephrologist, Department of Pediatric Nephrology, Dr. Sami Ulus Kadin Doğum Çocuk Sağliği ve Hastaliklari Eğitim ve Araştirma Hastanesi, Sağlik Bilimleri Üniversitesi, Ankara, Turkey.; VI MD. Physician and Pediatric Nephrologist, Department of Pediatric Nephrology, Dr. Sami Ulus Kadin Doğum Çocuk Sağliği ve Hastaliklari Eğitim ve Araştirma Hastanesi, Sağlik Bilimleri Üniversitesi, Ankara, Turkey.; VII MD. Physician and Pediatric Nephrologist, Department of Pediatric Nephrology, Dr. Sami Ulus Kadin Doğum Çocuk Sağliği ve Hastaliklari Eğitim ve Araştirma Hastanesi, Sağlik Bilimleri Üniversitesi, Ankara, Turkey.; VIII MD. Physician, Professor, Pediatric Nephrologist and Rheumatologist, Department of Pediatric Nephrology and Rheumatology, Dr. Sami Ulus Kadin Doğum Çocuk Sağliği ve Hastaliklari Eğitim ve Araştirma Hastanesi, Sağlik Bilimleri Üniversitesi, Ankara, Turkey.

**Keywords:** Child, Juvenile idiopathic arthritis, Tocilizumab, Childhood, Chronic arthritis, Interleukin-6 inhibitors

## Abstract

**BACKGROUND:**

Juvenile idiopathic arthritis (JIA) is the commonest chronic rheumatic disease among children. When not treated effectively, JIA can lead to functional disability, due to joint damage, along with long-term morbidities.

**OBJECTIVES:**

To describe the use of tocilizumab therapy for 11 patients with polyarticular JIA (pJIA) and systemic JIA (sJIA) who presented inadequate response or were refractory to disease-modifying anti-rheumatic drugs (DMARDs) and/or other biological therapies; and to evaluate its benefits, safety and tolerability.

**DESIGN AND SETTING:**

Observational retrospective case series at a tertiary-level training and research hospital.

**METHODS:**

We reviewed the medical records of 11 consecutive patients with JIA who received tocilizumab (anti-IL-6) therapy in our pediatric nephrology and rheumatology outpatient clinic. We analyzed their demographic data, clinical and laboratory findings, treatment response and adverse reactions. We determined the efficacy of tocilizumab treatment using the American College of Rheumatology (ACR) pediatric (Pedi) response criteria, including ACR Pedi 30, 50, 70 and 90 scores. We used the Wilcoxon test to compare measurements before and after treatment.

**RESULTS:**

Tocilizumab was given to seven patients with sJIA and four with pJIA (one of the pJIA patients was rheumatoid factor-positive). In most patients, we observed improvement of symptoms, absence of articular and extra-articular inflammation and continued inactive disease. ACR Pedi 30, 50 and 70 scores were achieved by 90.9% of the patients. Five patients showed minor side effects, possibly due to use of tocilizumab.

**CONCLUSIONS:**

Tocilizumab therapy should be considered for treating patients with diagnoses of pJIA or sJIA who are resistant to non-biological DMARDs and/or other biological therapies.

## INTRODUCTION

Juvenile idiopathic arthritis (JIA) is the most common chronic rheumatic disease in the pediatric population. JIA is characterized by unknown etiology and onset before the age of 16 years. Systemic JIA (sJIA) and polyarticular JIA (pJIA) are associated with increased joint damage, treatment refractoriness, prolonged course and poor outcome.^1^^,^^2^^,^^3^ pJIA is defined as arthritis in five or more joints during the first six months of the disease. sJIA is defined as arthritis and intermittent fever for two or more weeks, plus any of the following: typical rash, generalized lymphadenopathy, hepatosplenomegaly or serositis.^4^ Currently, sJIA is classified as a multifactorial autoinflammatory disease.^5^ Patients with these two subtypes of JIA generally have inadequate responses to non-steroidal anti-inflammatory drugs (NSAIDs) and non-biological disease-modifying anti-rheumatic drugs (DMARDs).^6^

IL-6 is a proinflammatory cytokine that plays an important role in the articular and extra-articular manifestations of JIA, as well as in the chronic complications of the disease.^7^ The clinical symptoms of sJIA are attributed to overproduction of IL-6.^8^ It is known that IL-6 increases in both the serum and the synovial fluid of patients with pJIA, and that the serum concentration of IL-6 is also positively correlated with the severity of joint involvement.^9^^,^^10^

Tocilizumab (an anti-IL-6 drug) is a recombinant, humanized monoclonal antibody that binds to IL-6 receptors and is commonly used for treating patients with active sJIA, alone or in combination with methotrexate.^11^ Inhibition of IL-6 signaling in response to tocilizumab can significantly improve the symptoms of sJIA. Phase II^12^^,^^13^ and phase III^14^ clinical trials that included children with sJIA showed significant reductions in inflammatory response, with improvement in osteoporosis and growth retardation.

Tocilizumab is an effective treatment that reduces the signs and symptoms of disease, and improves quality of life (QoL) and physical functioning in patients with pJIA.^15^ This drug is indicated for patients aged two or more years, for treating active sJIA and moderate to severe active pJIA, in situations of insufficient response to or intolerance of NSAIDs and DMARDs.

We aimed to describe the use of tocilizumab therapy for 11 patients with polyarticular JIA (pJIA) and systemic JIA (sJIA) who presented inadequate response or were refractory to disease-modifying anti-rheumatic drugs (DMARDs) and/or other biological therapies; and to evaluate its benefits, safety, and tolerability.

## METHODS

We retrospectively reviewed the medical records of patients diagnosed as presenting JIA who were followed up at the Pediatric Nephrology and Rheumatology Outpatient Clinic of Dr. Sami Ulus Çocuk Hospital, Ankara, Turkey, between September 2014 and October 2017. There were 11 JIA patients aged 3-18 years who were treated with tocilizumab. The tocilizumab treatment was administered via intravenous infusion: 8-10 mg/kg once every month for pJIA patients; and 8-12 mg/kg every 14 days for sJIA patients.

JIA was diagnosed based on the classification criteria of the International League of Associations for Rheumatology (ILAR).^16^ All the patients were treated in accordance with the standardized medication of the consensus-based treatment plans of the Childhood Arthritis and Rheumatology Research Alliance (CARRA) for pJIA and sJIA.^17^^,^^18^ These patients had presented treatment failure when other therapies (both biological and non-biological) had been used previously. Treatment failure was defined as an inadequate response or refractoriness or intolerance to other drugs that had been administered for at least three months.

Demographic data, clinical and laboratory findings, acute phase reactants, concomitant medications, response to treatment and adverse reactions were recorded in relation to all patients. All of them underwent laboratory analyses before tocilizumab treatment was started, along with evaluation for tuberculosis, including chest X-ray, tuberculin skin test (TST) and/or interferon-gamma release assay (IGRA).^19^ Cases of macrophage activation syndrome (MAS) were diagnosed based on clinical symptoms and laboratory findings, in accordance with the guidelines proposed by Ravelli et al.^20^

Disease activity was measured by means of the American College of Rheumatology pediatric response criteria (ACR Pedi), including ACR Pedi 30, 50, 70 and 90 scores.^14^^,^^15^ The clinical response was defined by using the core set, which include six markers, as follows: (1) number of joints with active arthritis; (2) functional ability; (3) number of joints with limited range of motion; (4) parent/patient’s overall assessment on a visual analogue scale (VAS) (scored on a 10-cm VAS); (5) physician’s overall assessment on a visual analogue scale (VAS); and (6) erythrocyte sedimentation rate. The ACR Pedi 30, 50 and 70 responses were defined as at least 30%, 50% and 70% improvement in three or more markers of the JIA core set, compared with the baseline, while no more than one of the remaining markers worsened by more than 30%.

Use of tocilizumab was started for treatment of signs and symptoms of active JIA in patients who had not shown any ACR Pedi 30 response and had responded inadequately to previous therapy with disease-modifying antirheumatic drugs, corticosteroids and other biological medications.

For the JIA patients who failed to respond to the current (or existing) treatment due to clinical unresponsiveness or toxicity to previous treatments, the treatment was changed to one using medication with a different mechanism of action. The treatment response was assessed in accordance with the definitions of the Classification and Response Criteria Subcommittee of the American College of Rheumatology Committee on Quality Measures.^21^

This retrospective study was approved by the ethics committee of Ankara Numune Training and Research Hospital (date: March 8, 2018; decision no: E. Kurul-E-18-1825). It was conducted in accordance with the principles of good clinical practice and the Declaration of Helsinki.

### Statistical analysis

Statistical analyses were performed using the SPSS software, version 20. The Kolmogorov-Smirnov test was used to assess the normality assumption for continuous variables. Non-normally distributed variables were presented as the median with the interquartile range (25^th^ to 75^th^ percentile). Categorical variables were presented as the number (with the percentage). The Wilcoxon test was used to compare the measurements from before and to after the treatment. P-values less than 0.05 were considered statistically significant.

## RESULTS

Among the 350 JIA patients in the hospital’s database, the case series consisted of 11 children with JIA (seven with sJIA and four with pJIA) who were treated with tocilizumab. At the onset of the disease, the patients were aged between 1 and 16 years (median: 4 years). At the onset of tocilizumab therapy, the patients were aged 3-18 years. The demographic data, clinical findings and treatment responses are shown in Table 1 and Table 2.

**Table 1.** Patients’ data and baseline characteristics

Patient characteristics	Value
Age in years at juvenile idiopathic arthritis onset, median (range)	4.2 (1-16)
Time in months that elapsed until administration of tocilizumab, median (range)	40.9 (6-150)
Sex, n (%)	
Male	4 (36.3)
Female	7 (63.7)
Subtype of juvenile idiopathic arthritis, n (%)	
Systemic	7 (63.7)
Polyarticular	4 (36.3)
Age in years at tocilizumab onset, median (interquartile range)	10 (5-14)
Disease duration in months, median (interquartile range)	32 (21-90)
Clinical findings at onset, n (%)	
Rash	6 (54.5)
Arthritis	11 (100)
Fever	7 (63.7)
Lymphadenopathy	5 (45.4)
Hepatomegaly	5 (45.4)
Splenomegaly	5 (45.4)
Macrophage activation syndrome	3 (27.2)
Number of joints with active arthritis at the start of tocilizumab therapy, median (interquartile range)	8 (1-18)
Number of joints with active arthritis after tocilizumab therapy, median (interquartile range)	0 (0)

**Table 2.** Patients’ characteristics and possible side effects associated with treatment

Patient number	Diagnosis	Sex	Age at diagnosis (years)	Disease duration (months)	Duration of tocilizumab use (months)*	Previous treatments	VAS1	VAS2	Adverse effect with tocilizumab
1	sJIA	M	9	15	5	NSAIDs-MTX-CS	10	2	
2	sJIA	F	10	58	31	NSAIDs-MTX-CS-CAN	10	3	
3	sJIA	F	5	28	12	NSAIDs-MTX-CS	10	0	
4	sJIA	F	4	21	12	NSAIDs-MTX-CS-CAN	10	0	
5	sJIA	F	13	32	13	MTX-CS-CAN	6	2	
6	sJIA	M	2	21	16	NSAIDs-CS	10	0	Diarrhea, fungal skin infection, nasopharyngitis, bronchitis
7	sJIA	F	4	90	37	NSAIDs-MTX-CS-Anti-TNF	10	3	
8	pJIA	F	3	118	17	NSAIDs-MTX-Anti-TNF	10	3	Nasopharyngitis
9	pJIA	M	1	70	17	NSAIDs-MTX-CS-Anti-TNF	8	2	Nasopharyngitis, epistaxis
10	pJIA	M	2	178	28	NSAIDs-MTX-CS-Anti-TNF	10	0	
11	pJIA	F	16	19	7	NSAIDs-MTX-CS-Anti-TNF	8	4	

*Duration of tocilizumab use: defined as the time from the beginning of the treatment with tocilizumab to the last dose of tocilizumab or to the time of writing this manuscript.

VAS1 = visual analogue scale before treatment with tocilizumab; VAS2 = visual analogue scale after treatment with tocilizumab.

sJIA = systemic juvenile idiopathic arthritis; pJIA = polyarticular juvenile idiopathic arthritis; DMARDs = disease-modifying antirheumatic drugs; CAN = canakinumab; NSAIDs = nonsteroidal anti-inflammatory drugs; MTX = methotrexate; CS = corticosteroids; Anti-TNF = anti-tumor necrosis factor; VAS = visual analogue scale.

In all the sJIA patients, the systemic signs of the disease (rash, arthritis, fever, lymphadenopathy, hepatomegaly and splenomegaly) completely disappeared after a few days, following injection of tocilizumab. This improvement was then maintained throughout the tocilizumab treatment.

Following this treatment, there were significant decreases in the median white blood cell count (P < 0.01) and platelet count (P = 0.021), and significant increases in the median hemoglobin level (P < 0.01) and mean platelet volume (P = 0.046) (Table 3). There were also statistically significant decreases in the median erythrocyte sedimentation rate (ESR) and the median C-reactive protein (CRP) level (P < 0.01 for each). Anemia was noted in seven patients (63.6%) before the treatment with tocilizumab, of whom four were sJIA patients. However, only one patient with pJIA had anemia following the treatment with tocilizumab. This patient was the only one who did not respond to this treatment.

**Table 3.** Laboratory analyses on children with juvenile idiopathic arthritis who were treated with tocilizumab

Parameters	At onset of tocilizumab use* Median (range)	After tocilizumab use* Median (range)	P-value
Hemoglobin, g/dl	11.3 (10.0-11.9)	12.5 (12.15-13.10)	< 0.01
White blood cells, 10^9^ cells/l	11.5 (10.1-17.5)	7.29 (5.43-8.70)	< 0.01
Granulocyte count	8.22 (6.92-10.19)	3.07 (2.29-4.88)	< 0.01
Lymphocyte count	2.3 (1.18-4.6)	2.47 (1.67-3.09)	0.859
Platelet count, 10^9^/l	457 (345-536)	278 (243-285)	0.021
Mean platelet volume, fl	7.3 (6.8-7.8)	7.9 (7.3-8.1)	0.046
Alanine aminotransferase level, U/l	12 (10-23)	15 (13-18)	0.895
C-reactive protein level (mg/l)*	45.6 (17.3-101)	3.1 (< 3.1)	< 0.01
Erythrocyte sedimentation rate (mm/hour)	42 (30-48)	3 (2-5)	< 0.01

*In our laboratory, the lowest measurable C-reactive protein (CRP) value was < 3.1 mg/l. CRP level after tocilizumab treatment was < 3.1 mg/l in all patients.

The pre-tocilizumab evaluation showed that three of the patients (one of them was a sJIA patient) were TST and IGRA-positive. Latent tuberculosis treatment (isoniazid) was given for six months. and the tests were negative after treatment with isoniazid. After treatment with tocilizumab, TST and IGRA tests on all 11 patients were negative and no tuberculosis was observed in any of the patients.

The results from pain assessments before tocilizumab treatment were the following: the median VAS on the parent/patient’s overall assessment of wellbeing was 10 cm (range 6-10 cm); and the median VAS on the physician’s overall assessment of disease activity was 10 cm (range 8-10 cm). Treatment with tocilizumab was associated with better parent/patient VAS and physician VAS: median of 2 cm (range 0-3) for each of them (P < 0.01).

Clinical remission was achieved following commencement of use of tocilizumab in all the sJIA patients but in only three of the four pJIA patients (75%). We observed that treatment with tocilizumab led to a decrease in the number of actively arthritic joints (Table 1). The median duration of use of tocilizumab was 16 months (range 12-28 months). Use of tocilizumab was discontinued in only one patient during this study, while 90.9% (10/11) of the patients continued to receive tocilizumab. Thus, in total, 90.9% of the patients who had not responded to earlier biological therapy achieved ACR Pedi 30, 50 and 70 scores through use of tocilizumab.

All of the patients had been treated with non-biological DMARDs and/or NSAIDs before starting to receive tocilizumab, as follows: methotrexate: n = 10 (i.e. all patients except for one with sJIA); NSAIDs: n = 10 (i.e. all patients except for one with sJIA); and corticosteroids: n = 10 (i.e. all patients except for one with pJIA). Some of the patients had been taking more than one type of medicine.

In addition, seven patients had previously also used another biological agent and one patient had previously used more than two biological agents. In total, four sJIA patients used only tocilizumab as biological therapy, and the other three sJIA patients switched to tocilizumab after having used canakinumab. For four pJIA patients, the biological treatment agent was switched from etanercept to tocilizumab. One of these patients was subsequently switched from tocilizumab to rituximab during the follow-up.

Among the ten patients who had taken corticosteroids at the baseline and during treatment with tocilizumab, eight (80%) discontinued their use of corticosteroids (three pJIA and five sJIA patients). Moreover, four patients (one of them presenting pJIA) did not receive any drugs concomitantly with tocilizumab. However, some patients continued to receive treatment with non-biological DMARDs after they started to receive tocilizumab: five used methotrexate (two of these were pJIA patients), three used NSAIDs (all of these were pJIA patients) and two used corticosteroids (one of these was a pJIA patient).

One patient who was seropositive for both rheumatoid factor and anti-cyclic citrulline peptide did not respond to tocilizumab treatment, which was used for seven months. This patient was subsequently switched to rituximab therapy. Unfortunately, in the case of this patient, there was also no successful response to rituximab therapy.

In all, five patients (45.4%) (one of these was a sJIA patient) experienced a range of possible minor adverse events. None of the patients were reported to have had uveitis before or after treatment with tocilizumab. One of the sJIA patients had diarrhea, but without any organisms isolated, and this patient additionally presented fungal skin infection, nasopharyngitis and bronchitis. Other adverse events observed included nasopharyngitis (three patients in total, of whom two were pJIA patients) and epistaxis (one pJIA patient). No anaphylaxis-like reactions developed in any of the patients and none of the patients had to discontinue tocilizumab due to side effects. None of the patients developed any infection requiring intravenous antibiotics or hospitalization, malignancy, autoimmune diseases, uveitis, high liver function test results, hypothyroidism, diverticulitis or kidney stones during their treatment with tocilizumab. None of the patients developed neutropenia. None of the patients developed amyloidosis and/or proteinuria, and none of the patients presented elevated cholesterol levels following treatment with tocilizumab. None of the patients died during this treatment.

Two patients had three episodes of macrophage activation syndrome (MAS) (in one patient, this occurred twice; and only one had bone marrow alterations), before the treatment with tocilizumab. However, MAS did not develop in any patients after the treatment with tocilizumab.

## DISCUSSION

The findings from the present retrospective observational case series showed that intravenous tocilizumab may be acceptable for treating sJIA and pJIA. The percentage of patients with sJIA-associated symptoms, such as fever, rash, lymphadenopathy, hepatomegaly, splenomegaly or arthritis, significantly decreased after treatment with tocilizumab. Clinical improvement was observed in the majority of the patients (90.9%) after treatment with tocilizumab, which is a higher success rate than previously reported.^22^^,^^23^^,^^24^^,^^25^^,^^26^ In the present study, in total, ACR Pedi 30, 50 and 70 scores were achieved in 10 (90.9%) of our JIA patients. Different ACR Pedi 30, 50 and 70 scores in response to use of tocilizumab have been reported in the literature.^14^^,^^15^^,^^22^^,^^24^^,^^25^^,^^26^

Adverse events were seen in 45.4% of our patients who used tocilizumab, including nasopharyngitis, diarrhea, skin fungal infection, nasopharyngitis, bronchitis and epistaxis, as previously reported.^22^^,^^24^ There was a decrease in the mean number of joints with active arthritis among our patients, during treatment with tocilizumab. Most of the patients (90.9%) did not have any joints with active arthritis after their treatment with tocilizumab, which was a much higher rate than had previously been reported in relation to sJIA and pJIA.^22^^,^^23^^,^^24^^,^^25^^,^^26^

The CRP and ESR levels decreased significantly in our patients after they started to receive treatment with tocilizumab. These levels then remained within normal limits throughout the therapy period, which was concordant with earlier reports.^22^^,^^23^^,^^24^^,^^25^^,^^26^ Use of corticosteroids was successfully tapered off through tocilizumab therapy, even though they could not be completely withdrawn in the cases of eight patients (80%). These findings were much better than those observed in some clinical trials.^27^^,^^28^

As also previously reported, we observed that the treatment with tocilizumab in our patients increased their hemoglobin levels and percentage lymphocyte counts, and decreased the neutrophil, platelet and white blood cell counts.^15^^,^^28^ Güneş et al.^29^ reported that there was a significant association between mean platelet volume (MPV) and disease activity in JIA patients. There was a statistically significant increase in MPV after the treatment with tocilizumab in our patients.

The major limitation of the present case series was its single-center retrospective design. The small number of patients, short follow-up and the absence of a control group limited the evaluation of the effect of treatment in this case series.

## CONCLUSIONS

This observational retrospective small series described patients with juvenile idiopathic arthritis that had been refractory to the usual treatment or had not responded to it. The present findings showed that the overall risk/benefit profile of tocilizumab used among JIA patients was acceptable, given the severity of the disease, the observed improvement in clinical symptoms and laboratory findings, and the possibility of tapering off the use of corticosteroids concomitantly with the treatment with tocilizumab.

Our findings are encouraging with regard to use of tocilizumab, as a possible viable alternative for JIA patients who have presented an inadequate response or been refractory to other therapies. Additional research is required to confirm the present findings and to determine the optimum duration of tocilizumab treatment in JIA patients.
